# Early lessons from schistosomiasis mass drug administration programs

**DOI:** 10.12688/f1000research.6826.1

**Published:** 2015-10-28

**Authors:** W. Evan Secor

**Affiliations:** 1 Parasitic Diseases Branch, Division of Parasitic Diseases and Malaria, Center for Global Health, Centers for Disease Control and Prevention, Atlanta, GA, 30329, USA

**Keywords:** schistosomiasis, mass drug administration, praziquantel, schistosome

## Abstract

Mass drug administration using praziquantel is the backbone of the current strategy for the control of schistosomiasis. As the theoretical plans have moved into practical application, certain challenges with this approach have surfaced, and it is likely that annual mass drug administration alone may not be sufficient to achieve program goals. However, mass drug administration is still the only available intervention that can be readily used in the wide variety of settings where schistosomiasis is endemic. The task then becomes how to improve this approach and identify what adjuncts to mass drug administration are effective, as programs move from morbidity control to elimination goals. Other aspects worthy of consideration include how best to employ new diagnostic tools to more easily identify where treatment is needed, and new formulations of praziquantel to extend the availability of treatment to all age groups. The aim of this review is to highlight both areas of challenge and of opportunity to improve the public health impact of schistosomiasis control programs.

## A new focus on schistosomiasis control and elimination

Over the last decade, there has been an increased emphasis on schistosomiasis control, especially with respect to using mass drug administration (MDA) to reduce its prevalence and the intensity of infections. This shift has largely been driven by the introduction of the preventative chemotherapy approach for neglected tropical diseases and the passage of World Health Assembly (WHA) resolutions 54.19 (2001; http://www.who.int/neglected_diseases/mediacentre/WHA_54.19_Eng.pdf?ua=1) and 65.21 (2012; http://www.who.int/neglected_diseases/mediacentre/WHA_65.21_Eng.pdf) that set control and elimination goals for schistosomiasis, respectively. In addition, access to treatment for those persons in need has dramatically increased as a result of aid agency purchase and manufacturer donation of praziquantel, the only drug currently available for the treatment of schistosome infections, along with efforts of groups such as the Schistosomiasis Control Initiative (SCI, http://www3.imperial.ac.uk/schisto) to work with ministries of health to distribute praziquantel ^1^. Finally, increased support for operational research on how best to distribute treatment has facilitated the posing of questions that have direct public health impact in a more widespread approach than was previously possible. While there have been clear public health benefits associated with schistosomiasis MDA in certain settings ^2, 3^, it has not been an unqualified success ^4– 6^. This review will focus on some of the practical questions that have surfaced with the introduction of MDA for schistosomiasis and issues that have implications for the successful implementation of schistosomiasis control and elimination programs.

## Challenges with providing MDA

Despite the dramatic expansion of praziquantel availability, data from 2013 indicate that of the more than 260 million people in need of treatment for schistosomiasis, less than 40 million received it ^7^. The shortfall can be attributed to a number of factors, including a remaining deficit (about 120 million treatments) in the amount of available praziquantel ^1^. Furthermore, even with significant price reduction or donation of the drug, the costs associated with identifying where MDA is needed and the delivery of the drugs create barriers for many national control programs in the absence of external funding to support these activities. Other obstacles include the lack of compliance with treatment programs by persons needing to take the drug. While health education increases community cooperation with praziquantel delivery programs, the side effects (fever, nausea, abdominal pain, diarrhea, fatigue) associated with dying worms, especially the first time someone is treated when worm burdens tend to be the highest, can lead to wariness about receiving follow-up treatments ^8– 11^. Further research on how best to promote participation in treatment campaigns, some of which will need to be tailored to specific countries, languages, or ethnic groups, is essential. MDA strategies are geared towards school age children but adults and pre-school aged children also contribute to ongoing transmission, suggesting that achieving elimination will require treating these other age groups as well. There is also a growing recognition that even very young children can become infected with schistosomes and suffer health consequences. However, current formulations of praziquantel are not appropriate for MDA in this age group because of the size and taste of the tablets. The therapeutic dose younger children need may also differ from that required by older individuals, as suggested by recent studies in Uganda ^12^. Fortunately, efforts are currently underway to develop a pediatric formulation of praziquantel and to define the dosing regimen. As this formulation becomes available, operational research on how to carry out treatments targeted to young children will be needed, in parallel with the development of treatment delivery approaches to improve compliance among school children and adults.

## Advances in determining where to treat

The initial requirement for any control program is to accurately determine the areas where treatment is needed. For schistosomiasis, this has traditionally been done by parasitologic assessment of stool or urine samples, depending on the schistosome species endemic in the area. Although parasitologic methods have recognized limitations in sensitivity, they were (until very recently) the only feasible option for estimating the prevalence of intestinal schistosomiasis and the only way to monitor the intensity of human infection for any of the species. As a result, the current World Health Organization (WHO) guidelines for schistosomiasis control are heavily dependent on detection of eggs in stool for *Schistosoma mansoni* and *S. japonicum* or urine for *S. haematobium* ^13^. Recently, a point of contact (POC) test that detects a *S. mansoni* carbohydrate antigen in the urine of infected individuals has become commercially available. This circulating cathodic antigen (CCA) POC test can indicate a relative intensity of infection and distinguish active infection, or reinfection, from cure following treatment. A large number of studies have evaluated the POC-CCA in comparison to stool examination by the Kato-Katz method and found that it is at least as good as traditional stool examination for mapping areas in need of MDA ^14– 19^. In general, the POC-CCA appears to be more sensitive than traditional stool examination methods but questions remain about whether disparities in results obtained when comparing the two methods are attributable to the known insensitivity of the Kato-Katz method or imperfect specificity of the POC-CCA test ^20^. However, when considering all the expenses associated with laboratory testing and treatment-associated expenditures, the costs of using either test are comparable ^19, 21^. Because the POC-CCA does not require equipment, it should be easier to deploy than the Kato-Katz method in areas that need mapping for *S. mansoni* prevalence. Nevertheless, training for POC-CCA use and interpretation will be needed and there is a distinct need to develop bench aids for this test. It is also not possible to simply apply the WHO guidelines that were written for morbidity control based on stool exam prevalence levels to the POC-CCA, which consistently detects higher prevalence levels. Thus, while the introduction of the POC-CCA is perhaps the most important technical advance for *S. mansoni* control since the release of praziquantel, more operational research is needed before it can achieve its full potential and be incorporated into WHO recommendations. The POC-CCA may also be useful for detecting *S. japonicum* infections ^22^.

The paradox of the POC-CCA test is that, even though it uses a urine sample for the assay, it is not a reliable test for *S. haematobium* infections. Fortunately, another carbohydrate, the circulating anodic antigen (CAA), is effective for detecting both urogenital and intestinal schistosomiasis ^23, 24^. It is also considered more sensitive and specific for *S. mansoni* than the POC-CCA. The drawback of the CAA test is that it involves equipment-requiring processing of samples prior to testing and the output is a non-visual signal that requires an automated reader. However, research is ongoing to develop the CAA into a more field applicable test that would have the advantage of detecting both intestinal and urinary schistosomiasis.

The detection of specific antibodies may also become important for control and elimination programs for schistosomiasis, although they are likely to be employed in different settings, or at different phases of the program than egg or antigen detection tools ^25^. An advantage of antibody assays is the ability to directly observe the collection of finger stick blood from the population being surveyed. Although urine collection is easier than stool collection, it is not feasible or culturally acceptable to directly observe the collection of either and therefore both present an increased risk of sample sharing. In addition, small quantities of blood can be used in multiplex assays that may simultaneously test for a variety of neglected tropical diseases, other infectious agents, and monitor vaccine coverage ^26^. Thus, a single sample can be used for several public health programs, thereby providing cost savings, compared to performing an independent survey for each infection or vaccine response of interest. Many low-cost rapid diagnostic tests are based on antibody detection and could be adapted for schistosomiasis, provided the proper antigen was selected. It is also theoretically possible to develop pan-schistosome or species-specific antigens depending on the intended use of the assay. A major drawback with using antibody-based assays is that the current antigens that are used for immunodiagnosis are recognized by host sera even after successful cure. Thus, it is not possible to distinguish former infections from active infections with great certainty, and therefore not possible to monitor decreases in prevalence levels as a control program progresses. Antibodies are also a less reliable indicator of intensity of infection than egg or antigen detection methods. However, antibody detection will likely be very useful for schistosomiasis elimination programs; children born after cessation of transmission would not be exposed to infection and thereby become very sensitive sentinels to confirm that elimination has been achieved. Further, as immunodiagnostic tests are developed that use individual antigens rather than antigen mixtures, immunoassays that are positive during active infection but become negative shortly after treatment may become available. One antigen that shows promise in this regard is recombinant SP-13 from *S. japonicum* ^27^. Detection of parasite DNA in stool or urine could also be a sensitive method for specifically identifying active infections ^28^.

## MDA plus what?

Current schistosomiasis control guidelines are based on different frequencies and target populations of MDA, with the most intensive intervention being annual MDA of all community members ^29^. This approach is predicated in large part on the benefits of annual MDA for reducing prevalence of lymphatic filariasis, onchocerciasis, and blinding trachoma ^30, 31^. However, growing evidence from schistosomiasis control efforts suggest that yearly MDA alone may not be sufficient to achieve program goals, especially when the objective is the elimination of transmission ^32– 34^. Research into what other interventions are both cost effective and environmentally acceptable is needed. A vaccine for schistosomiasis is quite desirable and the identification of novel vaccine targets that are responsible for vital biologic functions are providing intriguing new strategies to attack the parasite ^35– 37^. However, as has been the case for the last 3 decades, it seems that a vaccine for schistosomiasis is still 5–10 years and millions of dollars away from being a reality. Therefore, in the near term it is likely that other interventions involving water are the most likely adjuncts to MDA for reducing infection ^38, 39^.

An unfortunate side effect of the introduction and initial success of praziquantel was the assumption that treatment alone would be adequate to reach program goals. This belief contributed to reduced support for research into other control approaches. Because of the expense of water and sanitation systems, efforts to reduce urine or fecal contamination of water, as well as limiting exposure of people to contaminated water, will rely heavily on health education and behavioral modification. These approaches may need to be specifically tailored to individual communities and are therefore difficult to readily apply across endemic areas. The control of intermediate host snails can be highly effective but most interventions that have demonstrated success involve the introduction of molluscicides that also kill other aquatic species or entail the introduction of non-native snail predators, both of which have limited acceptability for local populations or groups with environmental concerns. One exciting idea that does not suffer from these limitations is represented by The Upstream Alliance project, which will reintroduce native *Macrobrachium vollenhoveni* prawns in the Senegal River upstream of the Daima Dam. Following completion of this dam in 1986, there was an outbreak of new schistosome infections that has in part been attributed to the interruption of the prawn’s ability to move up the river from its breeding grounds in brackish water. *M. vollenhoveni* are voracious predators of the schistosome intermediate host snails. The restriction of their migration led to an expansion of snail populations, which in turn led to increased transmission of schistosomiasis ^40^. The Upstream Alliance hopes to couple prawn aquaculture with the reintroduction of prawns above the dam to create an economically self-sustaining intervention to reduce schistosome infection prevalence ^41^. If successful, it will be a robust model for snail control programs in other areas with native *Macrobrachium spp.* populations.

## Adapting programs as they progress

In the “staged control” strategy for schistosomiasis, program objectives change from morbidity control to reduction of infection to elimination of transmission to post transmission surveillance, depending on the infection levels of the population ^42^. One of the biggest unknowns for schistosomiasis control is what prevalence cutoffs merit changes in treatment strategies and, in fact, what those different strategies should be as the goals of the program change. As mentioned above, the current WHO guidelines for schistosomiasis were developed at a time when praziquantel was less abundant and more expensive, and therefore the primary goal was the reduction of severe hepatosplenic disease for intestinal schistosomiasis and the prevention of bladder and kidney complications for urogenital schistosomiasis. Now that praziquantel is more readily available, it is recognized that treatment should be extended more generally, as it is not only those with the most severe manifestations of schistosomiasis that suffer morbidity. In fact, a strong case has been made that there is no such thing as an asymptomatic schistosome infection ^43^. Thus, there may really be no practical differences between reducing morbidity and reducing infection.

Monitoring of control program progress, like mapping, has traditionally been performed by measuring egg prevalence and the intensity of schistosome infections in school age children because they provide a useful barometer of the level of infection in other age groups in the community ^44^. This age group also tends to have the highest intensities of infection, so decreases in their egg output would result in fewer eggs that could contaminate fresh water and infect snails. Fewer infected snails would lead to the release of fewer infectious cercariae and a theoretical reduction in the “force of transmission”. A test to measure force of transmission in areas where people come into contact with water would be a more timely way to assess the impact of control efforts, rather than having to rely solely on measuring changes in infection levels in people. Unfortunately, previous attempts to measure the number of cercariae in water have not been successful. Sentinel mice and snail sampling, followed by cercarial shedding or PCR, have been somewhat informative but have not yet been incorporated as ways to monitor the impact of control programs. A method that has been somewhat successful in assessing water bodies for cercariae of avian schistosomes, ultrafiltration followed by real-time PCR, can detect as few as 5 cercariae in 100 liters of water ^45^. If this method will also work for detecting cercariae of schistosomes infectious for humans, control programs may have a technique to more directly assess the impact of different control efforts.

## Is praziquantel sufficient?

Another potential concern for treatment programs is the incomplete efficacy of praziquantel. The introduction of MDA for schistosomiasis has, fortunately, not resulted in evidence of widespread clinical resistance to drugs. However, it has long been recognized that a single treatment, especially for persons with high intensities of infection, is not adequate to kill all the worms. This finding has been further highlighted in recent studies comparing parasitologic and antigen detection assays following treatment ^46, 47^. Many individuals become egg negative but retain antigen positivity, suggesting that viable adult worms remain even if egg excretion has stopped. From a transmission perspective, and perhaps even from a morbidity standpoint, if egg laying stops and does not resume, the treatment has accomplished its goal. However, worms that are not killed may only be temporarily affected and subsequently resume egg laying. Interpretation of post treatment data is also complicated by the decreased efficacy of praziquantel against juvenile worms that can produce eggs once they mature. Provision of a second dose of praziquantel to target worms that may not have been killed by the first treatment produces increased cure rates and greater egg reduction than a single treatment ^48^. These studies highlight the need to continue research into improved formulations of praziquantel, if there are more effective dosing schedules than annual MDA depending on a location’s force of transmission or schistosomiasis species, as well as control *versus* elimination program goals. There is also concern that reliance on a single drug is risky, which argues for continued investigation into new or repurposed drugs for treatment ^49– 52^.

## Conclusions

The prospects for a global reduction in schistosomiasis prevalence and intensity are better now than ever before. Sub-Saharan Africa remains the biggest challenge, although even more developed countries like Brazil and China that have had control programs in place for many years still have much work remaining to achieve elimination. In addition to the WHA resolutions, the formation of groups such as SCI, the Schistosomiasis Consortium for Operational Research and Evaluation (SCORE), the Coalition for Operational Research on Neglected Tropical Diseases (COR-NTD) and, most recently, the Global Schistosomiasis Alliance (GSA) has provided better opportunities for researchers to interact with WHO, ministry of health officials, and schistosomiasis control program managers to identify and test practical solutions for challenges encountered where MDA has been used, and to begin to define and test the strategies for effecting and verifying elimination where appropriate. The pending completion of multi-year operational research studies should provide strong data for the development of updated evidenced-based guidelines for schistosomiasis in the near future. However, progress and answers will take time, requiring patience from donors and governmental aid agencies. Similarly, when prevalence levels decrease and schistosomiasis becomes a lower public health priority, ministries of health in endemic countries will need to maintain control activities amidst competing agendas to achieve elimination. Diagnostic tools with improved sensitivity and specificity, as well as operational research on how to employ them, are critical needs for elimination strategies in areas with decreasing prevalence and intensity of infection. Continued coordination of efforts, along with innovative thinking to identify effective interventions to complement MDA, will be necessary to reduce the public health burden and ultimately eliminate schistosomiasis.

## Abbreviations

CAA, circulating anodic antigen; CCA, circulating cathodic antigen; MDA, mass drug administration; POC, point of contact; WHA, World Health Assembly; WHO, World Health Organization.

## Disclaimer

The findings and conclusions in this report are those of the author and do not necessarily represent the official position of the Centers for Disease Control and Prevention.

## References

[1] Fenwick A: Praziquantel: do we need another antischistosoma treatment? *Future Med Chem.* 2015;7(6):677–80. 10.4155/fmc.15.16 25996058

[2] French MD Churcher TS Gambhir M: Observed reductions in *Schistosoma mansoni* transmission from large-scale administration of praziquantel in Uganda: a mathematical modelling study. *PLoS Negl Trop Dis.* 2010;4(11):e897. 10.1371/journal.pntd.0000897 21124888 PMC2990705

[3] Sesay S Paye J Bah MS: *Schistosoma mansoni* infection after three years of mass drug administration in Sierra Leone. *Parasit Vectors.* 2014;7:14. 10.1186/1756-3305-7-14 24401567 PMC3895768

[4] Lelo AE Mburu DN Magoma GN: No apparent reduction in schistosome burden or genetic diversity following four years of school-based mass drug administration in Mwea, central Kenya, a heavy transmission area. *PLoS Negl Trop Dis.* 2014;8(10):e3221. 10.1371/journal.pntd.0003221 25299057 PMC4191953

[5] Tuhebwe D Bagonza J Kiracho EE: Uptake of mass drug administration programme for schistosomiasis control in Koome Islands, Central Uganda. *PLoS One.* 2015;10(4):e0123673. 10.1371/journal.pone.0123673 25830917 PMC4382187

[6] Ross AG Olveda RM Li Y: An audacious goal: the elimination of schistosomiasis in our lifetime through mass drug administration. *Lancet.* 2015;385(9983):2220–1. 10.1016/S0140-6736(14)61417-3 25467574

[7] Schistosomiasis: number of people treated worldwide in 2013. *Wkly Epidemiol Rec.* 2015;90(5):25–32. 25638822

[8] Omedo M Ogutu M Awiti A: The effect of a health communication campaign on compliance with mass drug administration for schistosomiasis control in western Kenya--the SCORE project. *Am J Trop Med Hyg.* 2014;91(5):982–8. 10.4269/ajtmh.14-0136 25246690 PMC4228896

[9] Won KY Abudho B Blackstock AJ: Assessment of quality of life as a tool for measuring morbidity due to *Schistosoma mansoni* infection and the impact of treatment. *Am J Trop Med Hyg.* 2014;90(2):322–8. 10.4269/ajtmh.13-0361 24323511 PMC3919242

[10] Zwang J Olliaro PL: Clinical efficacy and tolerability of praziquantel for intestinal and urinary schistosomiasis-a meta-analysis of comparative and non-comparative clinical trials. *PLoS Negl Trop Dis.* 2014;8(11):e3286. 10.1371/journal.pntd.0003286 25412105 PMC4238982

[11] Muhumuza S Olsen A Katahoire A: Uptake of preventive treatment for intestinal schistosomiasis among school children in Jinja district, Uganda: a cross sectional study. *PLoS One.* 2013;8(5):e63438. 10.1371/journal.pone.0063438 23667617 PMC3646788

[12] Stothard JR Sousa-Figueiredo JC Betson M: Schistosomiasis in African infants and preschool children: let them now be treated! *Trends Parasitol.* 2013;29(4):197–205. 10.1016/j.pt.2013.02.001 23465781 PMC3878762

[13] World Health Organization: Helminth Control in School-Age Children: A Guide for Managers of Control Programmes. Geneva: World Health Organization;2011 Reference Source

[14] Coulibaly JT Knopp S N'Guessan NA: Accuracy of urine circulating cathodic antigen (CCA) test for *Schistosoma mansoni* diagnosis in different settings of Côte d'Ivoire. *PLoS Negl Trop Dis.* 2011;5(11):e1384. 10.1371/journal.pntd.0001384 22132246 PMC3222626

[15] Tchuem Tchuenté LA Kueté Fouodo CJ Kamwa Ngassam RI: Evaluation of circulating cathodic antigen (CCA) urine-tests for diagnosis of *Schistosoma mansoni* infection in Cameroon. *PLoS Negl Trop Dis.* 2012;6(7):e1758. 10.1371/journal.pntd.0001758 22860148 PMC3409114

[16] Navaratnam AM Mutumba-Nakalembe MJ Stothard JR: Notes on the use of urine-CCA dipsticks for detection of intestinal schistosomiasis in preschool children. *Trans R Soc Trop Med Hyg.* 2012;106(10):619–22. 10.1016/j.trstmh.2012.06.010 22858241

[17] Colley DG Binder S Campbell C: A five-country evaluation of a point-of-care circulating cathodic antigen urine assay for the prevalence of *Schistosoma mansoni*. *Am J Trop Med Hyg.* 2013;88(3):426–32. 10.4269/ajtmh.12-0639 23339198 PMC3592520

[18] Erko B Medhin G Teklehaymanot T: Evaluation of urine-circulating cathodic antigen (Urine-CCA) cassette test for the detection of *Schistosoma mansoni* infection in areas of moderate prevalence in Ethiopia. *Trop Med Int Health.* 2013;18(8):1029–35. 10.1111/tmi.12117 23590255

[19] Adriko M Standley CJ Tinkitina B: Evaluation of circulating cathodic antigen (CCA) urine-cassette assay as a survey tool for *Schistosoma mansoni* in different transmission settings within Bugiri District, Uganda. *Acta Trop.* 2014;136:50–7. 10.1016/j.actatropica.2014.04.001 24727052

[20] Foo KT Blackstock AJ Ochola EA: Evaluation of point-of-contact circulating cathodic antigen assays for the detection of *Schistosoma mansoni* infection in low-, moderate-, and high-prevalence schools in western Kenya. *Am J Trop Med Hyg.* 2015;92(6):1227–32. 10.4269/ajtmh.14-0643 25870418 PMC4458830

[21] Worrell CM Bartoces M Karanja DM: Cost analysis of tests for the detection of *Schistosoma mansoni* infection in children in western Kenya. *Am J Trop Med Hyg.* 2015;92(6):1233–9. 10.4269/ajtmh.14-0644 25870422 PMC4457354

[22] van Dam GJ Odermatt P Acosta L: Evaluation of banked urine samples for the detection of circulating anodic and cathodic antigens in *Schistosoma mekongi* and *S. japonicum* infections: a proof-of-concept study. *Acta Trop.* 2015;141(Pt B):198–203. 10.1016/j.actatropica.2014.09.003 25225158

[23] Corstjens PL De Dood CJ Kornelis D: Tools for diagnosis, monitoring and screening of *Schistosoma* infections utilizing lateral-flow based assays and upconverting phosphor labels. *Parasitology.* 2014;141(14):1841–55. 10.1017/S0031182014000626 24932595 PMC4265670

[24] Knopp S Corstjens PL Koukounari A: Sensitivity and Specificity of a Urine Circulating Anodic Antigen Test for the Diagnosis of *Schistosoma haematobium* in Low Endemic Settings. *PLoS Negl Trop Dis.* 2015;9(5):e0003752. 10.1371/journal.pntd.0003752 25973845 PMC4431728

[25] Solomon AW Engels D Bailey RL: A diagnostics platform for the integrated mapping, monitoring, and surveillance of neglected tropical diseases: rationale and target product profiles. *PLoS Negl Trop Dis.* 2012;6(7):e1746. 10.1371/journal.pntd.0001746 22860146 PMC3409112

[26] Lammie PJ Moss DM Brook Goodhew E: Development of a new platform for neglected tropical disease surveillance. *Int J Parasitol.* 2012;42(9):797–800. 10.1016/j.ijpara.2012.07.002 22846784

[27] Xu X Zhang Y Lin D: Serodiagnosis of *Schistosoma japonicum* infection: genome-wide identification of a protein marker, and assessment of its diagnostic validity in a field study in China. *Lancet Infect Dis.* 2014;14(6):489–97. 10.1016/S1473-3099(14)70067-2 24656567

[28] Meurs L Brienen E Mbow M: Is PCR the Next Reference Standard for the Diagnosis of *Schistosoma* in stool? A Comparison with Microscopy in Senegal and Kenya. *PLoS Negl Trop Dis.* 2015;9(7):e0003959. 10.1371/journal.pntd.0003959 26217948 PMC4517772

[29] World Health Organization: Schistosomiasis: progress report 2001–2011 and strategic plan 2012–2020. Geneva: World Health Organization;2013 Reference Source

[30] Ramaiah KD Ottesen EA: Progress and impact of 13 years of the global programme to eliminate lymphatic filariasis on reducing the burden of filarial disease. *PLoS Negl Trop Dis.* 2014;8(11):e3319. 10.1371/journal.pntd.0003319 25412180 PMC4239120

[31] Evans DS Alphonsus K Umaru J: Status of Onchocerciasis transmission after more than a decade of mass drug administration for onchocerciasis and lymphatic filariasis elimination in central Nigeria: challenges in coordinating the stop MDA decision. *PLoS Negl Trop Dis.* 2014;8(9):e3113. 10.1371/journal.pntd.0003113 25233351 PMC4169246

[32] Knopp S Stothard JR Rollinson D: From morbidity control to transmission control: time to change tactics against helminths on Unguja Island, Zanzibar. *Acta Trop.* 2013;128(2):412–22. 10.1016/j.actatropica.2011.04.010 21586268

[33] Njenga SM Mutungi FM Wamae CN: Once a year school-based deworming with praziquantel and albendazole combination may not be adequate for control of urogenital schistosomiasis and hookworm infection in Matuga District, Kwale County, Kenya. *Parasit Vectors.* 2014;7:74. 10.1186/1756-3305-7-74 24552246 PMC3936945

[34] Ross AG Olveda RM Chy D: Can mass drug administration lead to the sustainable control of schistosomiasis? *J Infect Dis.* 2015;211(2):283–9. 10.1093/infdis/jiu416 25070942

[35] Carvalho-Queiroz C Nyakundi R Ogongo P: Protective Potential of Antioxidant Enzymes as Vaccines for Schistosomiasis in a Non-Human Primate Model. *Front Immunol.* 2015;6:273. 10.3389/fimmu.2015.00273 26082781 PMC4451692

[36] You H Gobert GN Cai P: Suppression of the Insulin Receptors in Adult *Schistosoma japonicum* Impacts on Parasite Growth and Development: Further Evidence of Vaccine Potential. *PLoS Negl Trop Dis.* 2015;9(5):e0003730. 10.1371/journal.pntd.0003730 25961574 PMC4427307

[37] Li XH de Castro-Borges W Parker-Manuel S: The schistosome oesophageal gland: initiator of blood processing. *PLoS Negl Trop Dis.* 2013;7(7):e2337. 10.1371/journal.pntd.0002337 23936568 PMC3723592

[38] Evan Secor W: Water-based interventions for schistosomiasis control. *Pathog Glob Health.* 2014;108(5):246–54. 10.1179/2047773214Y.0000000149 25175875 PMC4153826

[39] Grimes JE Croll D Harrison WE: The roles of water, sanitation and hygiene in reducing schistosomiasis: a review. *Parasit Vectors.* 2015;8:156. 10.1186/s13071-015-0766-9 25884172 PMC4377019

[40] Savaya Alkalay A Rosen O Sokolow SH: The prawn *Macrobrachium vollenhovenii* in the Senegal River basin: towards sustainable restocking of all-male populations for biological control of schistosomiasis. *PLoS Negl Trop Dis.* 2014;8(8):e3060. 10.1371/journal.pntd.0003060 25166746 PMC4148216

[41] Sokolow SH Huttinger E Jouanard N: Reduced transmission of human schistosomiasis after restoration of a native river prawn that preys on the snail intermediate host. *Proc Natl Acad Sci U S A.* 2015;112(31):9650–5. 10.1073/pnas.1502651112 26195752 PMC4534245

[42] Engels D Chitsulo L Montresor A: The global epidemiological situation of schistosomiasis and new approaches to control and research. *Acta Trop.* 2002;82(2):139–46. 10.1016/S0001-706X(02)00045-1 12020886 PMC5633073

[43] King CH: It's time to dispel the myth of "asymptomatic" schistosomiasis. *PLoS Negl Trop Dis.* 2015;9(2):e0003504. 10.1371/journal.pntd.0003504 25695740 PMC4335065

[44] Mwinzi PN Muchiri G Wiegand RE: Predictive Value of School-Aged Children's Schistosomiasis Prevalence and Egg Intensity for Other Age Groups in Western Kenya. *Am J Trop Med Hyg.* 2015; pii: 15–0467. 10.4269/ajtmh.15-0467 26416108 PMC4674251

[45] Jothikumar N Mull BJ Brant SV: Real-time PCR and sequencing assays for rapid detection and identification of avian schistosomes in environmental samples. *Appl Environ Microbiol.* 2015;81(12):4207–15. 10.1128/AEM.00750-15 25862226 PMC4524150

[46] Mwinzi PN Kittur N Ochola E: Additional Evaluation of the Point-of-Contact Circulating Cathodic Antigen Assay for *Schistosoma mansoni* Infection. *Front Public Health.* 2015;3:48. 10.3389/fpubh.2015.00048 25853117 PMC4365547

[47] Lamberton PH Kabatereine NB Oguttu DW: Sensitivity and specificity of multiple Kato-Katz thick smears and a circulating cathodic antigen test for *Schistosoma mansoni* diagnosis pre- and post-repeated-praziquantel treatment. *PLoS Negl Trop Dis.* 2014;8(9):e3139. 10.1371/journal.pntd.0003139 25211217 PMC4161328

[48] King CH Olbrych SK Soon M: Utility of repeated praziquantel dosing in the treatment of schistosomiasis in high-risk communities in Africa: a systematic review. *PLoS Negl Trop Dis.* 2011;5(9):e1321. 10.1371/journal.pntd.0001321 21949893 PMC3176745

[49] Cioli D Pica-Mattoccia L Basso A: Schistosomiasis control: praziquantel forever? *Mol Biochem Parasitol.* 2014;195(1):23–9. 10.1016/j.molbiopara.2014.06.002 24955523

[50] Lee EF Fairlie WD: Repurposing apoptosis-inducing cancer drugs to treat schistosomiasis. *Future Med Chem.* 2015;7(6):707–11. 10.4155/fmc.14.164 25996064

[51] Ramamoorthi R Graef KM Dent J: Repurposing pharma assets: an accelerated mechanism for strengthening the schistosomiasis drug development pipeline. *Future Med Chem.* 2015;7(6):727–35. 10.4155/fmc.15.26 25996066

[52] Gelmedin V Dissous C Grevelding CG: Re-positioning protein-kinase inhibitors against schistosomiasis. *Future Med Chem.* 2015;7(6):737–52. 10.4155/fmc.15.31 25996067

